# The Role of Hysteroscopy in Reproductive Medicine and Subfertility: A Critical Narrative Review

**DOI:** 10.3390/medicina62102005

**Published:** 2026-10-16

**Authors:** Orestis Tsonis, Ioannis Paschopoulos, Kasmiria Ioanna Paschopoulou, Evangelia Deligeoroglou, Zoi Barmpalia, Minas Paschopoulos

**Affiliations:** 1 Fertility Preservation Service, Assisted Conception Unit, Guy’s and St Thomas’ NHS Foundation Trust, London SE1 9RT, UK; 2 Department of Obstetrics and Gynaecology, University Hospital of Ioannina, 455 00 Ioannina, Greece; 3 School of Medicine, Faculty of Health Sciences, National and Kapodistrian University of Athens, 115 27 Athens, Greece; 4 School of Medicine, Faculty of Health Sciences, University of Ioannina, 451 10 Ioannina, Greece; 5 Unit for Human Reproduction, Medical School, Aristotle University of Thessaloniki, 1st Department of Obstetrics & Gynecology, Papageorgiou General Hospital, 564 03 Thessaloniki, Greece

**Keywords:** hysteroscopy, subfertility, assisted reproduction, endometrial polyp, submucous fibroid, intrauterine adhesions, septate uterus, chronic endometritis

## Abstract

*Background and Objectives*: Hysteroscopy permits direct assessment and treatment of the uterine cavity, but diagnostic yield does not necessarily translate into improved fertility. This narrative review examines its role in subfertility and reproductive medicine. *Materials and Methods*: The original literature search covered PubMed/MEDLINE, Scopus and the Cochrane Library through February 2026. Targeted checks during revision added the 2026 chronic endometritis consensus and the full CERM trial report; these updates do not represent a new comprehensive search. Evidence was interpreted by study design, population, outcome and precision, without formal risk-of-bias assessment or meta-analysis. *Results*: Large randomised trials do not support routine hysteroscopy before first IVF or after repeated IVF failure when ultrasonography is normal. Polypectomy has evidence of improved pregnancy rates in women planning IUI, although spontaneous pregnancies contributed to that result and live-birth benefit remains uncertain. Hysteroscopic myomectomy may improve clinical pregnancy in women with cavity-distorting fibroids; evidence for live birth is less secure. Adhesiolysis restores cavity anatomy, but reproductive estimates largely derive from observational series. The small TRUST trial did not demonstrate a live-birth advantage from septum resection and cannot establish equivalence. Hysteroscopy may guide biopsy for chronic endometritis but does not replace histological assessment. CERM found no improvement with doxycycline in its composite of cumulative live births and ongoing pregnancies at ≥24 weeks among women with recurrent miscarriage and CD138-defined endometritis. Fertility-sparing endometrial cancer treatment requires oncological selection, progestin therapy and histological surveillance. *Conclusions*: Hysteroscopy should address a defined diagnostic or therapeutic question. Decisions should distinguish spontaneous conception, IUI and IVF/ICSI and acknowledge uncertainty in live-birth outcomes.

## 1. Introduction

The World Health Organization defines infertility as failure to achieve pregnancy after 12 months or more of regular unprotected sexual intercourse [1]. The term does not imply irreversible loss of reproductive potential; counselling should reflect the couple’s prognosis rather than the label alone. WHO estimates indicate that approximately 17.5% of adults experience infertility during their lifetime, or around one in six people worldwide [1]. Its consequences extend beyond treatment burden to psychological distress and the social and financial effects of delayed parenthood.

Uterine factors are potentially treatable contributors to subfertility, although estimates of their prevalence vary with definitions, population and diagnostic method [2]. Endometrial polyps, cavity-distorting fibroids, intrauterine adhesions and uterine septa are common reasons for further cavity assessment. Their presence should prompt a clinical question about symptoms or reproductive prognosis, rather than an assumption that every finding requires surgery [3].

Hysteroscopy provides direct visualisation of the uterine cavity and can combine diagnosis with treatment [4,5]. Miniaturised instruments and vaginoscopic techniques have extended its use in outpatient care. Saline distension supports cavity assessment and is compatible with mechanical instruments and bipolar energy, although safe operative practice still requires monitoring of fluid absorption [6,7]. Subtle findings such as micropolyposis, hyperaemia and stromal oedema may be visible, but their interpretation requires clinical context and, where chronic endometritis is suspected, histological assessment [8,9,10].

The dual diagnostic and therapeutic nature of hysteroscopy is relevant when an intracavitary lesion is suspected but incompletely characterised by transvaginal ultrasound (TVUS) or hysterosalpingography (HSG) [11]. Direct assessment can permit a see-and-treat procedure in appropriately selected women [5,12]. Diagnostic accuracy and fewer attendances are useful service outcomes, but neither should be presented as proof of improved implantation or live birth.

**Figure 1:**
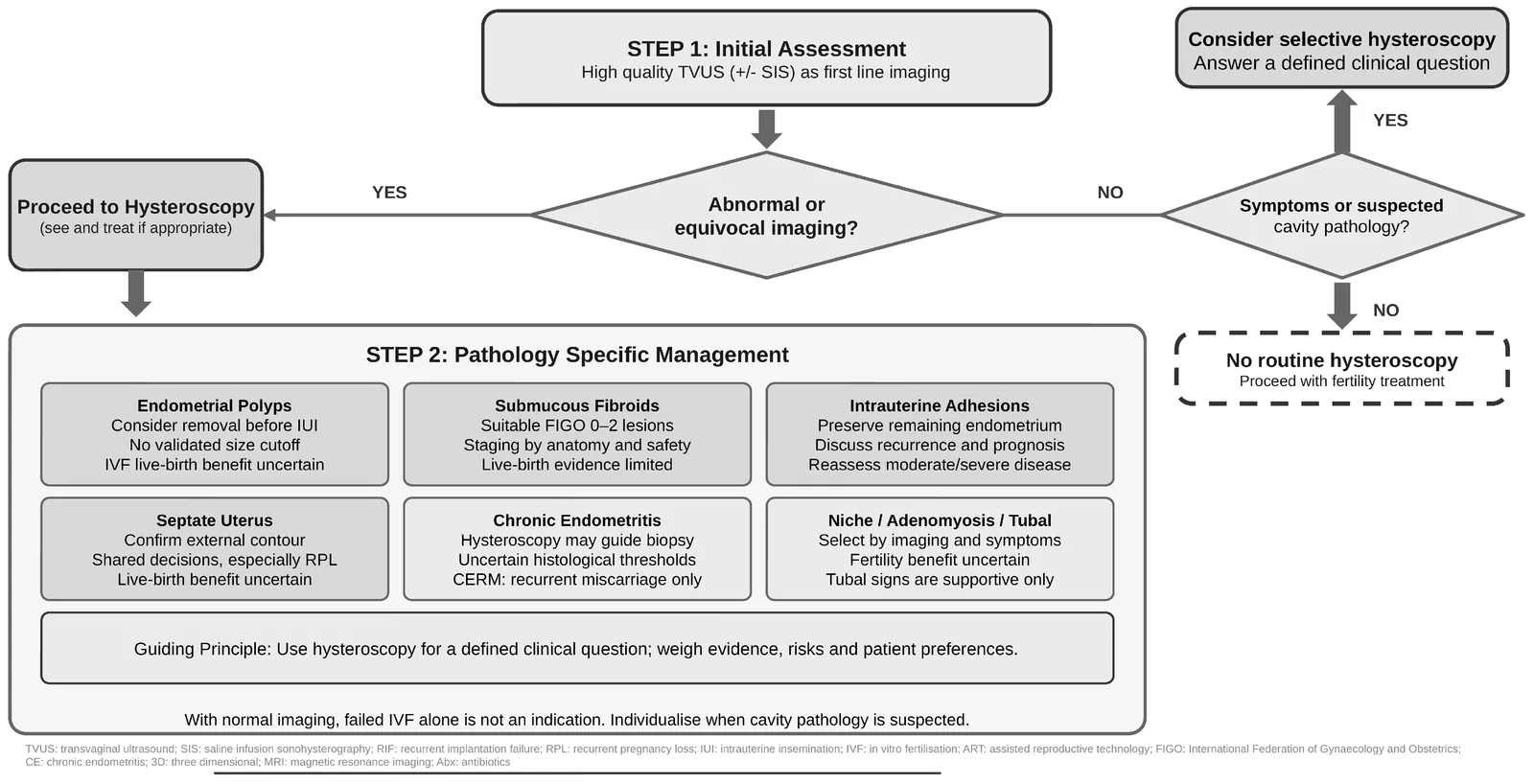
Clinical decision algorithm for hysteroscopy in the fertility pathway.

The evidence for treating identified pathology must be separated from the evidence for screening an apparently normal cavity. Meta-analyses have reported higher clinical pregnancy or live-birth rates after screening hysteroscopy, but these findings combine populations and trials of differing methodological quality [13,14,15]. In contrast, the large inSIGHT and TROPHY randomised trials did not demonstrate improved live birth before first IVF or after two to four failed IVF cycles when ultrasonography was normal [16,17]. We therefore favour selective investigation guided by imaging, symptoms and a specific clinical question. Table 1 summarises practical principles, Table 2 sets out pathology-specific decisions, and Table 3 distinguishes the populations and outcomes of pivotal trials. Figure 1 translates this approach into a clinical pathway.

Figure 1 is embedded above and provided separately in high-resolution PNG and vector PDF formats. The algorithm summarises the clinical approach discussed in Section 3 and Section 4; it is not a validated prediction rule.

## 2. Materials and Methods

This article is a narrative clinical review. The original manuscript’s search covered PubMed/MEDLINE, Scopus and the Cochrane Library through February 2026. Searches combined hysteroscopy terms with infertility, subfertility, assisted reproduction, IVF, IUI, implantation failure, recurrent pregnancy loss and reproductive outcomes. Pathology-specific terms included polyps, fibroids, intrauterine adhesions, septate uterus, chronic endometritis, caesarean scar niche, adenomyosis and tubal patency. Reference lists of relevant reviews and guidelines supplemented electronic searching.

Selection was directed by relevance to the clinical questions, rather than by a protocol-defined exhaustive screening process. The review was not registered, and formal risk-of-bias assessment, duplicate independent screening and quantitative pooling were not undertaken. English-language clinical literature formed the principal evidence base. The available search description does not constitute a reproducible historical database strategy, and screening counts are not presented. To make the revision transparent, Supplementary Material S1 supplies the exact PubMed queries executed for targeted verification on 5 October 2026, separately from the historical search. These checks added the chronic endometritis consensus and full CERM report published after the original endpoint, and the literature relevant to the reviewers’ requested corrections. Conference abstracts are not used to support treatment recommendations; the CERM abstract has been replaced by the full report.

### 2.1. Evidence Interpretation

Evidence was weighed by the question addressed, not by design labels alone. For intervention effects, directly applicable randomised trials with live-birth outcomes were given greatest weight. Systematic reviews were interpreted in light of the quality and applicability of their component studies; pooling small or biased trials did not override a large, relevant trial. Observational studies informed associations, uncommon harms and outcomes where trials were unavailable, but were not treated as proof of treatment benefit. Guidelines informed clinical practice while their underlying evidence was considered separately. Expert consensus informed definitions and areas of uncertainty, not causal treatment claims.

When findings conflicted, we compared population, comparator, outcome definition, follow-up and precision. Large trials in women with normal imaging were not extrapolated to women with documented lesions, and pregnancy outcomes were not substituted for live birth. The tables use three descriptive evidence categories: randomised evidence, observational evidence and guideline/consensus. These identify the basis of a statement and are not formal GRADE ratings. Imprecision, indirectness and heterogeneity are described in the interpretation column.

### 2.2. Reproductive Outcome Definitions

Clinical pregnancy denotes pregnancy confirmed by ultrasonography or definitive clinical signs; ongoing pregnancy is pregnancy continuing beyond a stated gestational threshold; live birth denotes birth with signs of life. Miscarriage is pregnancy loss before the study’s specified threshold and should usually be expressed per pregnancy, rather than per treated woman. Time to conception requires a defined starting point, follow-up and treatment of censoring [45]. Study-specific definitions are retained where they differ. We distinguish outcomes per randomised woman, analysed woman, pregnancy, treatment cycle or embryo transfer. Spontaneous conception, IUI and IVF/ICSI are separated where the source permits; mixed or unreported conception routes are explicitly labelled. Maternal and obstetric outcomes, including haemorrhage, preterm birth and placentation disorders, are distinct from fertility outcomes. Table 3 and Table 4 report denominators, follow-up and these interpretative limits.

## 3. Principles and Practice of Hysteroscopy

Hysteroscopy practice has evolved towards patient-centred outpatient care. The vaginoscopic approach avoids routine use of a speculum and tenaculum, and current RCOG guidance supports its use where feasible [6]. Unnecessary cervical dilatation should be avoided. Small-calibre rigid or flexible instruments permit diagnostic assessment and selected operative procedures. Normal saline is appropriate for diagnostic hysteroscopy, mechanical surgery and bipolar electrosurgery; monopolar electrosurgery requires a compatible electrolyte-free medium [7]. The medium, pressure and anticipated duration should be planned with the procedure and the woman’s comorbidities in mind.

Preprocedural counselling should explain likely discomfort, alternatives, available analgesia and the option to pause or stop. Pain varies considerably; oral analgesia alone should not be assumed sufficient for every woman. Local anaesthesia, sedation or theatre-based care may be appropriate according to preference, anticipated complexity and previous experience [6]. Services should audit completion, pain, patient experience and complications.

Diagnostic and operative hysteroscopy can both be undertaken in an office setting when the procedure is suitable and the woman wishes to proceed. More complex lesions or anticipated intolerance may require theatre-based care with an appropriate anaesthetic. Thus, “operative” does not necessarily mean general anaesthesia, and the setting should follow patient needs, procedural risk and available expertise [6,12]. Micropolyposis suggestive of chronic endometritis warrants assessment and possible biopsy, rather than indiscriminate excision.

Several key anatomical landmarks are essential for orientation and safe surgical intervention during hysteroscopy. The anterior, posterior, and lateral uterine walls, the endocervical canal, and both tubal ostia should be systematically inspected and clearly documented in every procedure [5,12]. High-quality still images or video recordings of the uterine cavity, accompanied by a structured report of intrauterine findings, facilitate accurate communication between clinicians and support multidisciplinary decision-making. Such documentation is particularly important within fertility services, where shared interpretation of findings directly influences treatment planning and patient outcomes.

Complications include vasovagal reactions, cervical trauma, perforation, bleeding, infection and excessive absorption of distension fluid. Risks differ between diagnostic assessment and complex surgery. In a prospective Dutch multicentre study of 13,600 procedures, complications requiring intervention occurred in 0.13% of diagnostic and 0.95% of operative procedures [49]. These historical estimates are not contemporary guarantees and should be supplemented by local audit; a single-centre retrospective series cannot define universal complication rates [50]. Monitoring fluid deficit and agreeing stopping limits are essential even with saline, which avoids hypotonic hyponatraemia but can still cause fluid overload [7]. Pain or loss of orientation should prompt reassessment rather than forced entry.

Blood loss and haematological status also matter in women referred with abnormal uterine bleeding, particularly before operative treatment of polyps or submucosal fibroids. Jobda et al. retrospectively studied 543 women and reported postoperative haemoglobin in the study-defined moderate-anaemia range in 90 women (16.54%); this category used haemoglobin 10–12 g/dL and was most frequent with heavy menstrual bleeding [51]. The postoperative timing and mixed-age population limit extrapolation to preoperative anaemia in fertility patients. NICE recommends a full blood count for women with heavy menstrual bleeding [19]. In this subgroup, assessment and treatment of anaemia, and planning for anticipated operative blood loss, are reasonable components of preprocedural care.

## 4. Clinical Indications of Hysteroscopy in Reproductive Medicine

Hysteroscopy is most useful when direct cavity assessment or treatment is likely to change management. Abnormal or equivocal imaging, abnormal uterine bleeding, suspected adhesions after instrumentation and a clearly identified intracavitary lesion are established reasons to consider it [3,12]. Reproductive failure can prompt reassessment of the uterine cavity, but repeated miscarriage or failed transfer alone does not establish that routine hysteroscopy will improve live birth.

The clinical value of treatment differs across polyps, submucous fibroids, adhesions and septa. Anatomical restoration, menstrual improvement, clinical pregnancy and live birth are distinct outcomes; evidence for one should not be substituted for another [3]. Hysteroscopy also permits targeted endometrial sampling when a finding raises concern for chronic endometritis, hyperplasia or malignancy. Endometrial sampling should address a defined indication and be interpreted alongside imaging and clinical history [8,9].

For IVF/ICSI, normal imaging and absence of symptoms do not justify routine screening hysteroscopy. inSIGHT found no live-birth advantage before first IVF, and TROPHY found none after two to four unsuccessful IVF cycles [16,17]. ESHRE advises that hysteroscopy can be considered in recurrent implantation failure when a uterine abnormality is suspected; this is not a recommendation to scope after a fixed number of unsuccessful transfers [18]. Endometrial scratching is a separate intervention. In the PIP trial, scratching before fresh or frozen embryo transfer did not improve live birth; it should not be added routinely to hysteroscopy or IVF [44]. These conclusions concern the tested populations and do not exclude targeted treatment of documented pathology.

### 4.1. Endometrial Polyps

Endometrial polyps are focal overgrowths of glands and stroma. Reported frequency varies substantially with age, bleeding symptoms, referral setting and diagnostic method [21,52]. Most are benign, but abnormal bleeding and relevant malignancy risk factors affect the need for assessment. Excision, when undertaken, should provide tissue for histology [21]. A fertility indication should be considered separately from a bleeding or oncological indication.

Polyps may be incidental or present with abnormal uterine bleeding. Proposed reproductive mechanisms include interference with sperm transport and changes in endometrial receptivity, but these mechanisms do not establish a live-birth benefit from removing every small lesion [21,22]. The significance of an asymptomatic polyp therefore depends on symptoms, cavity involvement, planned treatment and the consequences of delaying conception or ART.

Polypectomy may be performed in the office or theatre, depending on the lesion and patient preference. In the randomised study by Pérez-Medina et al., 215 women with ultrasound-detected polyps planning IUI were allocated to polypectomy or diagnostic hysteroscopy with polyp biopsy. The evaluable analysis reported 64/101 versus 29/103 pregnancies, with a relative risk of 2.1 (95% CI 1.5–2.9) [23]. Follow-up encompassed the interval before treatment and up to four IUI cycles; 65% of pregnancies in the polypectomy group occurred before the first IUI. This result should therefore not be labelled an IUI-only effect or a live-birth outcome. Reviews support considering polypectomy in women planning IUI, while recognising the limitations of this single trial [3,22]. For IVF/ICSI, observational reports of benefit do not establish a live-birth advantage or a validated size threshold [22,53]. There is no adequate basis for a universal 5–7 mm intervention rule. Management of a small asymptomatic polyp should be individualised and should avoid unnecessary treatment delay.

### 4.2. Uterine Fibroids

Uterine fibroids are common benign smooth-muscle tumours; prevalence varies with age, ancestry and the method of ascertainment [54]. The FIGO system classifies their relationship to the endometrium and serosa [55]. Types 0–2 are submucosal, type 3 is entirely intramural but abuts the endometrium, and types 4–7 are intramural or subserosal. This anatomical distinction is more useful for fertility decisions than a general estimate of fibroid prevalence. Hysteroscopic treatment is directed chiefly at submucosal lesions; non-cavity-distorting intramural and subserosal fibroids require separate consideration [56,57].

Cavity distortion is the central reproductive concern. ASRM considers that hysteroscopic myomectomy for cavity-distorting myomas can improve clinical pregnancy rates, but evidence is insufficient to establish an effect on live birth or early pregnancy loss [24]. For non-cavity-distorting intramural fibroids, associations with poorer outcomes do not by themselves establish benefit from surgery. A universal 4–5 cm fertility threshold is therefore inappropriate. Removal of an asymptomatic subserosal fibroid solely to improve fertility is not supported [24,56,57]. Obstetric risks associated with fibroids should be discussed separately from the probability of conception.

FIGO type 0 and type 1 fibroids, and selected type 2 lesions, are suitable for hysteroscopic myomectomy. Feasibility depends on size, intramural extension, location, cavity access and operator experience [25,26]. A staged procedure may be necessary when safe complete removal is limited by fluid deficit, visibility or myometrial involvement; this is a surgical safety decision rather than a proven fertility intervention or an automatic rule for lesions exceeding 3 cm. Reports of hysteroscopic treatment of type 3 lesions describe specialist techniques and do not establish routine indications or reproductive superiority [58]. Bipolar energy and mechanical approaches are compatible with saline, but saline does not eliminate fluid overload [7]. Cold-loop techniques may reduce thermal exposure, although comparative live-birth evidence is limited.

Counselling should distinguish incomplete removal, need for a staged procedure, development of new fibroids and recurrence of symptoms. The balance of benefit and harm should be based on cavity involvement, symptoms and fertility plans, with realistic discussion of uncertainty in live-birth outcomes [3,24,56]. Repeated procedures also carry a risk of intrauterine adhesions and may delay fertility treatment.

### 4.3. Intrauterine Adhesions (Asherman Syndrome)

Intrauterine adhesions are fibrous bands within the cavity. Asherman syndrome describes adhesions accompanied by symptoms, such as menstrual disturbance, pain or reproductive difficulty; the terms are not exact synonyms [27,59]. Injury to the endometrial basalis, particularly after pregnancy-related instrumentation, is a common antecedent. Other causes include intrauterine surgery and selected infections [59,60,61]. Presentation ranges from incidental adhesions to hypomenorrhoea, amenorrhoea, subfertility or pregnancy loss, and symptom severity need not precisely mirror the anatomical extent.

Hysteroscopy is the reference method for diagnosing and treating intrauterine adhesions because it identifies their distribution and permits division under direct vision [27]. The operative aim is to restore the cavity while preserving remaining endometrium. Mechanical scissors can minimise thermal exposure; dense disease may require other instruments and specialist expertise. Comparative evidence does not establish a universally superior technique for reproductive outcomes [27,28,62]. Severe or recurrent disease warrants realistic counselling about endometrial function, repeat procedures and pregnancy prognosis.

Adhesion recurrence remains a concern after surgery. Some studies show that mechanical barriers and hyaluronic-acid gels can reduce reformation, but an improved adhesion score does not necessarily indicate improved live birth [27,29]. Postoperative oestrogen is widely used; the optimal regimen and incremental reproductive benefit remain uncertain. Planned reassessment, often by hysteroscopy, should take account of initial severity and recurrence risk, particularly after moderate or severe adhesions [27,63]. Adjuncts should be presented with their evidence limits rather than as a standard package guaranteed to restore fertility.

Reported pregnancy and live-birth outcomes after adhesiolysis vary with severity, age, baseline endometrial damage, conception method and follow-up [28,64]. Most data are uncontrolled or retrospective, so before-and-after improvement cannot reliably quantify the causal benefit of surgery. General ranges without a defined denominator and follow-up are not used here. A matched retrospective cohort also reported increased adverse obstetric outcomes after treated adhesions; placentation and other pregnancy complications must be distinguished from fertility outcomes [30]. Women who conceive should have their surgical history communicated to the obstetric team. Stem-cell and other regenerative treatments remain investigational [62].

### 4.4. Congenital Uterine Anomalies

A septate uterus is a congenital cavity anomaly that may be associated with pregnancy loss and adverse obstetric outcomes. Definitions differ between classification systems, affecting who is labelled as having a septum and who is offered surgery [31,65]. Both the cavity and external fundal contour should be assessed, preferably with three-dimensional TVUS, with saline infusion when appropriate; MRI can clarify selected uncertain cases. Hysteroscopy alone cannot reliably distinguish a septate from a bicornuate uterus [31]. Septum length or completeness should not be treated as a proven predictor of surgical benefit.

When surgery is chosen, hysteroscopic septum incision aims to improve cavity configuration while avoiding excessive fundal injury. Technique depends on anatomy and operator experience. Scissors and energy-based approaches are described, but reports of laser platforms or postoperative cavity appearance do not establish superiority for live birth [66,67,68]. The indication should be agreed before treatment; resection of an incidental or borderline variant should not be justified by anatomy alone [31].

Observational studies and their meta-analyses suggest lower miscarriage rates or better reproductive outcomes after septum surgery, but selection, regression to the mean and changes in subsequent fertility care may explain part of the association [65,69,70]. In TRUST, live birth following conception within 12 months occurred in 12/39 women after resection and 14/40 with expectant management (RR 0.88, 95% CI 0.47–1.65) [32]. This small trial did not demonstrate benefit, but its imprecision does not establish equivalence or exclude benefit or harm in particular subgroups. It was not adequately powered to resolve outcomes by septum subtype. ASRM supports shared decision-making for septum incision in recurrent miscarriage and acknowledges uncertainty for infertility [31]. Recurrent implantation failure alone should not be presented as a proven indication. Discussion should include the uncertain live-birth benefit, perforation and adhesion risks, and the alternative of expectant management.

### 4.5. Subtle Lesions and Chronic Endometritis

Chronic endometritis (CE) is usually described histologically by endometrial stromal plasma cells, commonly assessed with CD138 immunostaining. Diagnostic thresholds and their reproductive significance vary between studies and laboratories [71,72]. Hysteroscopic features that raise suspicion include hyperaemia, stromal oedema, micropolyps and a strawberry appearance. In a series of 2675 outpatient hysteroscopies, Tsonis et al. evaluated these features against CD138-based histology; polyps alone were not a reliable marker of CE [8]. Diagnostic reviews found substantial heterogeneity and support hysteroscopy as a complementary investigation rather than a replacement for biopsy [9,10]. The 2026 modified e-Delphi consensus recognises these features but remains expert consensus, not evidence that screening or treatment improves live birth [33]. It should inform terminology and diagnostic interpretation without overriding direct randomised evidence on treatment outcomes.

Reported CE prevalence depends strongly on referral population, sampling and diagnostic threshold [73,74]. CD138 can stain cells other than morphologically defined plasma cells; the count, tissue area or high-power-field denominator, morphology and threshold should therefore be reported [72]. Menstrual phase also influences detection [75]. Sampling timing should be specified and consistent with the diagnostic protocol, but current evidence does not support prescribing one phase as universally correct. Routine biopsy-based screening in otherwise asymptomatic women should not be inferred from diagnostic associations.

Evidence that antibiotic treatment of CE improves reproductive outcomes is uncertain and population-dependent. The full CERM randomised report, published after the original search endpoint, supersedes its conference abstract [34]. It recruited women aged 18 to <42 years with at least two consecutive first-trimester miscarriages and luteal-phase CD138-defined CE and compared doxycycline 100 mg twice daily for 14 days with placebo. Its primary outcome was cumulative live birth plus ongoing pregnancies at ≥24 weeks at trial cessation, not live birth alone. This occurred in 113/219 versus 109/219 women (adjusted RR 1.02, 95% credible interval 0.85–1.21). The trial counted all CD138-positive stromal cells, rather than only morphologically defined plasma cells, and stopped at interim analysis; follow-up ended in October 2023 [34,76]. The result does not support this doxycycline regimen to improve the composite reproductive outcome in the tested recurrent-miscarriage population. It should not be extrapolated to all infertility, IVF/ICSI, other antibiotic regimens or clinically apparent infection. Decisions about CE testing and treatment require discussion of uncertain thresholds, indication and evidence.

### 4.6. Fertility-Sparing Treatment of Endometrial Carcinoma

Fertility-sparing treatment is an oncological exception to definitive surgery, not routine treatment for women wishing to conceive. The ESGO/ESHRE/ESGE guideline identifies the usual candidate as a woman desiring pregnancy with grade 1 endometrioid carcinoma confined to the endometrium, without myometrial invasion or extrauterine disease, who can adhere to close surveillance [43]. Diagnosis should be reviewed by an experienced gynaecological pathologist, and imaging should assess myometrial invasion and adnexal or extrauterine disease. Counselling should address recurrence, progression, delayed definitive surgery and the possibility that fertility will not be achieved. Management requires a gynaecological oncology and reproductive medicine team.

Hysteroscopy provides directed tissue sampling and assessment of the lesion. In selected focal disease, resection can remove the visible tumour and provide separate samples from adjacent endometrium and underlying myometrium, while preserving the remainder of the cavity. Sampling must establish suitability for conservative management; it cannot substitute for staging assessment. The guideline supports hysteroscopic resection followed by progestin therapy, while recognising that comparative oncological and reproductive evidence is largely non-randomised [43,47]. Resection alone should not be presented as adequate definitive oncological treatment.

Progestin-based treatment uses a 52 mg levonorgestrel intrauterine system, oral progestins or a combined approach according to specialist assessment. Guideline oral regimens include megestrol acetate 160–320 mg/day or medroxyprogesterone acetate 400–600 mg/day [43]. Response must be defined histologically: a complete response means a negative endometrial biopsy, not simply a normal hysteroscopic appearance. The guideline expects complete response within 6–12 months, with decisions about non-response made in the specialist team. Earlier systematic reviews of progestin treatment demonstrate that an initial response does not ensure durable remission [46,77]. Grade 2 disease has been studied in small selected series, including the review by Ronsini et al.; those findings do not justify extending routine eligibility beyond the usual grade 1 population [48].

Oncological response, recurrence and reproduction require separate denominators. A meta-analysis of hysteroscopic-resection-based conservative treatment included 407 women with endometrioid cancer and 444 with atypical hyperplasia [47]. For cancer, pooled complete response was 88.6%; recurrence was 18.3% among responders. The pooled live-birth proportion was 26.0% among all treated women in the reproductive analysis, 30.6% among complete responders and 51.9% among responders who attempted conception. These estimates come from different subsets, not a single prospective cohort or a randomised comparison, and cannot be converted into raw counts. Follow-up varied, and a uniform time-to-conception estimate or clean separation of spontaneous conception from ART was unavailable. Table 4 presents the denominators and uncertainty alongside the earlier Gallos review [46]. Atypical hyperplasia and carcinoma outcomes should not be combined.

Surveillance requires endometrial histology, commonly by hysteroscopic biopsy at 3 and 6 months. Two consecutive complete-response biopsies at least 3 months apart are recommended before attempting pregnancy; further sampling every 3–6 months continues until pregnancy or definitive surgery [43]. Once response is confirmed, prompt reproductive planning can limit unnecessary delay; ART may be appropriate to shorten time to conception but should not be represented as guaranteeing pregnancy. Maintenance progestin treatment may be used when pregnancy is deferred. Definitive hysterectomy is recommended after completion of childbearing, and for progression, recurrence or failure to respond within the agreed treatment interval. Exceptional repeat conservative treatment requires specialist reassessment and explicit counselling. Oncological safety remains the first priority throughout.

### 4.7. Beyond Structural Pathology

A caesarean scar niche may be associated with postmenstrual bleeding or secondary subfertility. TVUS, with saline infusion when needed, assesses the defect and residual myometrial thickness. Hysteroscopy can define cavity involvement, but the choice of hysteroscopic, laparoscopic or combined treatment depends on anatomy, symptoms and pregnancy plans. Observational improvements after repair do not establish a causal live-birth benefit or a universal threshold for surgery [35,36]. For adenomyosis, expert TVUS and MRI remain the principal diagnostic approaches. Hysteroscopy may assess superficial changes or enable targeted sampling in selected cases, but it does not establish the extent of diffuse myometrial disease or a proven fertility benefit from excision [37,38,39,40]. Hysteroscopic bubble or flow signs can provide supportive information about tubal patency but should not replace HSG, HyCoSy or appropriately indicated laparoscopy when management depends on tubal status [41,42].

Routine cavity instrumentation or saline flushing should not be described as a fertility treatment in women with normal imaging. Apparent improvement in uncontrolled studies may reflect spontaneous conception, selection or concurrent treatment rather than the procedure itself [14,16,17]. The clinical decision is whether a defined finding is likely to change management enough to justify the discomfort, procedural risks and potential delay. Table 2 summarises these decisions and their evidence limits; Figure 1 provides a concise pathway.

## 5. Limitations and Future Directions

This review has limitations inherent to its narrative methodology. Author-directed selection may introduce selection and citation bias; language restriction and non-exhaustive retrieval may omit relevant studies. Without formal risk-of-bias assessment, the evidence categories used here cannot be interpreted as validated certainty ratings. Targeted revision searches improve coverage of specific questions but do not make the review comprehensive. Heterogeneity in populations, lesion definitions, techniques, concomitant treatment, outcome denominators and follow-up limits cross-study comparison. These limitations are particularly important when observational reproductive improvement is used to suggest benefit from surgery.

Future studies should use prospectively specified outcomes, including live birth per woman or couple, time to conception and patient-reported experience. Spontaneous conception, IUI and IVF/ICSI should be reported separately, and postoperative pregnancy risks should be captured independently from fertility outcomes. Adequately powered multicentre trials and linked registries could clarify which lesions benefit from intervention. Office hysteroscopy services should audit pain, completion, adverse events and treatment delay [20]. New imaging, miniaturised devices and artificial-intelligence tools require validation against appropriate diagnostic standards and clinically meaningful outcomes before routine adoption.

The practical priority is selective hysteroscopy that answers a clear clinical question. Better visualisation and technically successful surgery are valuable but do not guarantee a live birth. Decisions should combine the woman’s symptoms, imaging, reproductive history and preferences with the strength and limits of the evidence. Table 4 summarises outcomes of fertility-sparing cancer treatment, where oncological control and reproduction must be evaluated separately. Across indications, the aim is to avoid unnecessary intervention while ensuring access to appropriate treatment for clinically important pathology.

## 6. Conclusions

Hysteroscopy should be used selectively to answer a defined cavity question. Large trials do not support routine screening with normal imaging before IVF, including after repeated failure. Evidence for pregnancy benefit after treating selected lesions should not be restated as proven live-birth benefit. Septum surgery requires shared decisions and acknowledgement of imprecision. CE assessment requires histological context; CERM findings apply to its recurrent-miscarriage population and tested regimen. Fertility-sparing cancer care requires oncology selection, progestins, surveillance and a plan for definitive surgery.

## Figures and Tables

**Table 1:** Applied clinical principles for integrating hysteroscopy into fertility pathways.

Domain	Applied Clinical Advice and Evidence Basis
Initial assessment	Use expert TVUS, with SIS when needed. Consider hysteroscopy for abnormal or uncertain imaging or a defined symptom/lesion question. Normal imaging before first IVF or after repeated IVF failure does not support routine screening [16,17,18].
Communication and consent	Explain the expected diagnostic or therapeutic benefit, discomfort, alternatives and uncertainty in live birth. Reproductive failure alone is not proof of a correctable cavity lesion [6,18].
Technique and safety	Use vaginoscopy where feasible. Match distension medium to equipment, monitor fluid deficit, and stop in the case of unsafe visibility, pain or loss of orientation. Assess bleeding and anaemia in women with HMB [6,7,19].
When to hysteroscope	A suspected polyp, cavity-distorting fibroid, adhesion or other intracavitary lesion may warrant direct assessment. In RIF, consider it when a uterine abnormality is suspected, without a fixed transfer-count threshold [3,18].
Service design	Choose office versus theatre care by procedure complexity, patient preference and analgesia needs. Selected operative procedures can be performed in the office [6,12].
Training and audit	Document cavity findings and monitor pain, completion and adverse events. New technology should be evaluated against appropriate diagnostic standards and reproductive outcomes [6,20].

TVUS, transvaginal ultrasound; SIS, saline infusion sonohysterography; RIF, recurrent implantation failure; HMB, heavy menstrual bleeding; IVF, in vitro fertilisation.

**Table 2:** Pathology-specific decisions and evidence limits in reproductive hysteroscopy.

Pathology or Scenario	Decision and Evidence Limits
Endometrial polyps	Consider removal for symptoms or a justified reproductive indication. Pregnancy benefit in women planning IUI comes mainly from one RCT that included spontaneous conceptions. IVF/ICSI live-birth benefit and a 5–7 mm cutoff are unproven. Send excised tissue for histology [21,22,23].
Cavity-distorting fibroids	Consider hysteroscopic myomectomy for type 0/1 and suitable type 2 lesions. Evidence supports clinical pregnancy more than live birth. Staging follows anatomy and safety limits, without an automatic 3 cm fertility rule [7,24,25,26].
Intrauterine adhesions	Treat symptomatic disease under direct vision with endometrial preservation. Discuss recurrence, adjunct limitations and planned reassessment. Reproductive evidence is mostly observational; pregnancy after repair also warrants attention to obstetric risk [27,28,29,30].
Septate uterus	Confirm anatomy including external contour. Use shared decision-making, particularly for recurrent miscarriage. Infertility benefit is uncertain, and RIF alone is not a proven indication. TRUST did not establish equivalence [31,32].
Suspected chronic endometritis	Hysteroscopic features can guide biopsy, not establish CE alone. Specify histological criteria and sampling timing. CERM concerns doxycycline in recurrent miscarriage with CD138-defined CE, not all infertility or antibiotic treatment [8,9,33,34].
Caesarean scar niche	Assess residual myometrial thickness and symptoms on imaging; choose repair route with specialist input. Reproductive benefit and surgical thresholds remain uncertain [35,36].
Adenomyosis	Use expert TVUS/MRI for diagnosis. Hysteroscopy may address selected superficial findings or sampling; benefit from excision for fertility is unproven [37,38,39,40].
Tubal patency	Treat bubble/flow signs as supportive. Use formal HyCoSy/HSG or appropriately indicated laparoscopy when tubal status changes management [41,42].
Fertility-sparing endometrial cancer care	Select usual grade 1 endometrioid, non-invasive disease through oncology review. Combine appropriate hysteroscopic assessment/resection with progestins, histological surveillance and a plan for definitive surgery [43].

IUI, intrauterine insemination; IVF/ICSI, in vitro fertilisation/intracytoplasmic sperm injection; FIGO, International Federation of Gynecology and Obstetrics; CE, chronic endometritis; RIF, recurrent implantation failure; TVUS, transvaginal ultrasound; MRI, magnetic resonance imaging; HyCoSy, hysterosalpingo-contrast sonography; HSG, hysterosalpingography.

**Table 3:** Pivotal randomised reproductive trials: populations, outcomes and interpretation.

Study/Evidence Category	Population and Conception Setting	Outcome, Denominator and Follow-Up	Result and Interpretation
inSIGHT [16] Randomised	Normal TVUS; first IVF. A total of 750 women randomised.	Ongoing pregnancy >12 weeks resulting in live birth within 18 months. Source analysis: 369 versus 373 women.	209/369 vs. 200/373; RR 1.06 (95% CI 0.93–1.20). No demonstrated live-birth benefit from screening; not a trial of women with known lesions.
TROPHY [17] Randomised	Normal ultrasound; two to four failed IVF cycles; women <38 years.	Live birth (>24 weeks in trial definition) in the subsequent IVF treatment cycle, per randomised woman.	102/350 vs. 102/352; RR 1.00 (95% CI 0.79–1.25). No demonstrated benefit; failed cycles alone do not justify routine hysteroscopy.
PIP [44] Randomised	IVF with fresh or frozen embryo transfer; endometrial scratching versus no scratching.	Live birth in the trial treatment period, per randomised woman.	180/690 vs. 176/674; adjusted OR 1.00 (95% CI 0.78–1.27). Scratching is distinct from hysteroscopy and is not supported routinely.
Pérez-Medina [23] Randomised	Women with polyps planning IUI. Spontaneous pregnancies before IUI contributed.	Pregnancy before and through up to four IUI cycles; evaluable women 101 vs. 103 from 215 randomised.	64/101 vs. 29/103 pregnancies; RR 2.1 (95% CI 1.5–2.9). Pregnancy outcome, not live birth. Not an IUI-only estimate.
TRUST [32] Randomised	Septate uterus with subfertility, pregnancy loss or preterm birth history. Conception routes not separately estimated here.	Live birth (>24 weeks) following conception within 12 months; 39 vs. 40 analysed women.	12/39 vs. 14/40; RR 0.88 (95% CI 0.47–1.65). Small and imprecise; absence of demonstrated benefit is not equivalence.
CERM [34] Randomised; targeted 2026 update	≥2 consecutive first-trimester miscarriages; CD138-positive stromal-cell definition of CE. Not an IVF/ICSI-specific population.	Composite cumulative live births plus ongoing pregnancy ≥24 weeks at trial cessation; 219 per arm. Follow-up ended October 2023.	113/219 vs. 109/219; adjusted RR 1.02 (95% credible interval 0.85–1.21). No improvement with the tested doxycycline regimen; not a pure live-birth estimate.

RR, relative risk; OR, odds ratio; CI, confidence interval. CERM reports a Bayesian credible interval. Counts use the source’s analysed denominator; the text identifies any difference from the randomised population. Mixed conception settings are not interpreted as route-specific effects.

**Table 4:** Fertility-sparing endometrial cancer evidence: oncological and reproductive outcomes.

Source/Evidence Category	Population and Treatment	Oncological and Reproductive Outcomes	Denominators and Interpretative Limits
Gallos et al. [46] Observational-study meta-analysis	A total of 408 endometrial cancer and 151 atypical complex hyperplasia cases in 34 studies; fertility-sparing treatment.	Cancer: pooled regression 76.2%, relapse 40.6%, live birth 28%. Hyperplasia reported separately in the source.	Pooled study-specific proportions. Relapse follows response; conception route and follow-up vary. Live birth is not a per-transfer rate or a guaranteed probability for a woman attempting pregnancy.
Zhao et al. [47] Observational-study meta-analysis	A total of 407 cancer and 444 atypical hyperplasia patients in 21 articles; hysteroscopic-resection-based conservative therapy. Cancer results shown separately.	Cancer: complete response 88.6%. Recurrence 18.3% among 327 responders (18 studies). Live birth: 26.0% (95% CI 17.3–35.5) among 358 treated women; 30.6% (21.0–41.0) among 307 responders; 51.9% (37.1–66.6) among 131 responders attempting conception.	Live-birth subsets: 18, 18 and 12 studies, respectively. Pooled proportions, not raw counts. Follow-up varies and conception routes are mixed. Non-randomised comparisons cannot establish treatment superiority.
Ronsini et al. [48] Small observational series synthesis	Selected stage IA grade 2 cancer; 103 patients across five studies.	Oncological response and recurrence varied with treatment and follow-up. No uniform reproductive estimate used here.	Indirect for usual grade 1 eligibility. Limited, selected data do not justify routine grade 2 fertility-sparing treatment.
ESGO/ESHRE/ESGE [43] Guideline	Usual candidate: grade 1 endometrioid cancer confined to the endometrium, no extrauterine disease.	Confirm histological response; monitor recurrence. Reproductive planning follows response, with definitive surgery after childbearing or for treatment failure/progression.	Clinical recommendations based mainly on non-randomised evidence. Surveillance and oncological safety are not fertility endpoints.

ART, assisted reproductive technology. Pooled proportions from different subsets are not directly comparable and cannot be multiplied by an overall sample size to obtain event counts. Follow-up and conception methods vary between component studies.

## Data Availability

No new data were created or analysed in this study. Data sharing is not applicable to this article.

## Supplementary Materials

The following supporting information can be downloaded at: https://www.mdpi.com/article/10.3390/medicina62102005/s1. Supplementary Material S1, “Targeted revision search and verification record”, is provided as a separate supplementary file. It documents the targeted PubMed verification searches executed on 5 October 2026 and is not a comprehensive or reconstructed historical literature search.
