# Supplementary material for: The Role of Hysteroscopy in Reproductive Medicine and Subfertility: A Critical Narrative Review
Source: Medicina (Kaunas). 2026 Oct 16;62(10):2005. doi: 10.3390/medicina62102005 (PMC13706842; doi:10.3390/medicina62102005)
Supplement: Supplementary file 1 [file medicina-62-02005-s001.zip › medicina-4589753-supplementary.pdf]

Supplementary Material S1

Targeted revision search and verification record

Manuscript: medicina-4589753. Original narrative search endpoint: February 2026. Revision checks: 5 October 2026. This record documents targeted PubMed queries used to verify pivotal trials and requested topics; it is not a reconstructed historical search strategy, exhaustive update, PRISMA flow diagram or screening log. The counts below are database query returns, not numbers screened or included.

Pivotal IVF and scratching trials

(hysteroscopy[Title/Abstract] OR "endometrial scratching"[Title/Abstract]) AND (TROPHY[Title/Abstract] OR inSIGHT[Title/Abstract] OR "A Randomized Trial of Endometrial Scratching before In Vitro Fertilization"[Title])

Executed: 2026-10-05. PubMed query returns: 21.

Septum evidence

("septum resection"[Title/Abstract] OR "uterine septum"[Title/Abstract]) AND (randomized[Title/Abstract] OR guideline[Title])

Executed: 2026-10-05. PubMed query returns: 32.

Chronic endometritis targeted updates

"chronic endometritis"[Title/Abstract] AND ("expert consensus"[Title/Abstract] OR doxycycline[Title/Abstract]) AND 2026[Date - Publication]

Executed: 2026-10-05. PubMed query returns: 14.

Fertility-sparing cancer evidence

("endometrial carcinoma"[Title/Abstract] OR "endometrial cancer"[Title/Abstract]) AND ("fertility-sparing"[Title/Abstract] OR "fertility sparing"[Title/Abstract]) AND (hysteroscop*[Title/Abstract] OR guideline[Title] OR "systematic review"[Title])

Executed: 2026-10-05. PubMed query returns: 137.

Anaemia source

35328147[uid]

Executed: 2026-10-05. PubMed query returns: 1.

Polyp trial

15760959[uid]

Executed: 2026-10-05. PubMed query returns: 1.

Additional verification

Article identity and citation metadata were checked by exact DOI/PMID against PubMed/Europe PMC records and, where required, Crossref. Pivotal trial abstracts and available full texts, the cancer guideline and hysteroscopic-treatment meta-analysis, the CE consensus, fluid guideline and Jobda study were inspected for population, outcome and denominator. Official WHO, RCOG and NICE documents were used for corporate references. These targeted checks do not imply independent duplicate extraction or full-text assessment of every paper in the narrative review.

Unrecoverable historical detail

The supplied search account lists PubMed/MEDLINE, Scopus and the Cochrane Library and the search concepts, but does not provide archived exact historical strings or screening counts. These have not been invented. Narrative selection and the resulting risk of selection/citation bias are described in Sections 2 and 5. The later 2026 CE consensus and full CERM report are identified explicitly as targeted updates.
